# THE ROLE OF GENDER AND POLYSUBSTANCE USE IN ABUSIVE BEHAVIORS EXHIBITED BY DEMENTIA FAMILY CAREGIVERS

**DOI:** 10.1093/geroni/igae098.0499

**Published:** 2024-12-31

**Authors:** Jessica Hernandez, Wesley Browning, Mustafa Yildiz, Vicki Winstead, Carolyn Pickering

**Affiliations:** The University of Texas Health Science Center, Houston, Texas, United States; The University of Texas Health Science Center Houston, Houston, Texas, United States; The University of Texas Health Houston, Houston, Texas, United States; The University of Texas Health Science Center, Houston, Texas, United States; The University of Texas Health Houston, Houston, Texas, United States

## Abstract

This study investigates the impact of polysubstance use on the likelihood of abusive and neglectful behaviors among dementia family caregivers, with a particular focus on how these relationships differ by gender. Utilizing Generalized Linear Mixed Models (GLMM), we analyzed data from 453 caregivers, assessing substance use and behavioral outcomes measured daily over a 21-day period. Our findings reveal that polysubstance use significantly increases the risk of both psychological and physical aggressive behaviors. When examining the interaction effects, we observed that the combination of being male and engaging in polysubstance use was particularly associated with a heightened risk of these behaviors, underscoring the importance of considering gender in caregiver assessments. The final models, refined through a stepwise selection process, incorporated key covariates such as caregiving hours, relationship type, emotional regulation difficulties, caregiver age, and ethnicity. These models offered a more comprehensive picture, showing that the dynamics of the caregiver/care-recipient relationship and the caregiver’s emotional regulation capabilities were significant factors influencing the likelihood of neglect and psychological aggression. In contrast, gender significantly modulated the association between polysubstance use and physical aggression. These nuanced insights highlight the complex interplay between caregiver demographics, substance use behaviors, and the potential for harmful caregiving practices. Our study emphasizes the need for targeted interventions that address the specific stressors and risks associated with male caregivers, as well as the broader implications for support programs that account for the multifaceted challenges faced by dementia family caregivers.

